# Changes in Hospitalizations at US Safety-Net Hospitals Following Medicaid Expansion

**DOI:** 10.1001/jamanetworkopen.2021.14343

**Published:** 2021-06-30

**Authors:** Karen E. Lasser, Zhixiu Liu, Meng-Yun Lin, Michael K. Paasche-Orlow, Amresh Hanchate

**Affiliations:** 1 Boston University School of Medicine, Boston, Massachusetts; 2 Section of General Internal Medicine, Boston Medical Center, Boston, Massachusetts; 3 Boston Medical Center, Boston, Massachusetts; 4 Boston University School of Public Health, Boston, Massachusetts; 5 Department of Social Sciences and Health Policy, Wake Forest School of Medicine, Winston-Salem, North Carolina

## Abstract

This cross-sectional study examines whether inpatient utilization among patients with lower socioeconomic status and among those who belong to racial/ethnic minority groups changed differentially in states that expanded Medicaid following the Patient Protection and Affordable Care Act (ACA).

## Introduction

Studies of Medicaid expansion under the Patient Protection and Affordable Care Act (ACA)^1,2^ have had conflicting findings regarding safety-net hospital (SNH) utilization and have not examined racial/ethnic differences in SNH use. We used data with a larger number of states; substantial racial/ethnic minority populations, including nearly 83% of the national Hispanic population; and with a longer period of observation. We hypothesized that inpatient utilization among patients with lower socioeconomic status and among those who belong to racial/ethnic minority groups would change differentially in Medicaid expansion states, as patients who previously did not have insurance might transfer care from SNHs to non-SNHs, which have more resources and greater access to specialty care.^3^

## Methods

The institutional review boards at Wake Forest and Boston University Schools of Medicine approved this study and granted a waiver of informed consent because it would not be feasible to obtain consent from the hospitalized patients whose data appear in this study. This study followed the Strengthening the Reporting of Observational Studies in Epidemiology (STROBE) reporting guideline for cross-sectional studies.

We used 2012 to 2017 all-payer inpatient discharge data^4,5^ from 11 Medicaid expansion states (Arkansas, Arizona, California, Colorado, Iowa, Illinois, Kentucky, New Jersey, New York, Oregon, and Pennsylvania; with the exception of 2017 data for Arkansas and New York) and 6 nonexpansion states (Florida, Georgia, North Carolina, Texas, Virginia, and Wisconsin) (eMethods and eFigure in the Supplement) and classified hospitals that appeared in the top quartile of Medicaid and uninsured discharges in 2012 in each state as SNHs. We grouped Medicaid-covered and uninsured hospitalizations for each quarter in each state into younger (age 26-64 years; target beneficiaries of expansion) and older (age ≥65 years) adults. Our outcome was the percentage of uninsured or Medicaid-insured hospitalizations in an SNH, overall and among subgroups by race/ethnicity and zip code–level poverty, based on the federal poverty level. We analyzed race data to capture unmeasured social factors (eg, structural racism, racial discrimination) and to identify disparities in utilization of safety-net hospitals. While race was not routinely self-identified in our data, race and ethnicity are separately identified in virtually all the states, which is the preferred approach. We used the combined race/ethnicity indicator developed by the Agency of Healthcare Research and Quality. Across all 17 states, the percentage of observations with missing data on race/ethnicity from 2012 to 2016 ranged from 0% to 5.8%, with a median of 1.7%.

We used a 3-way difference-in-differences study design, contrasting the change in the percentage of uninsured or Medicaid-insured hospitalizations to an SNH from the pre- to postexpansion periods between (1) expansion and nonexpansion states and (2) younger and older adults. Comparison of younger and older adults within geographic areas allows adjustment for unobserved temporal changes in patterns of hospital utilization. We estimated linear regression models with state-level fixed effects.^6^ We conducted analyses with Stata version 16.1 (StataCorp). Significance was set at *P* < .05, and all tests were 2-tailed. We provide further methodological details in the eMethods in the Supplement.

## Results

Overall, there were 60 632 753 discharges in the sample, with 42 343 336 (69.8%) among patients aged 65 years or older. We assumed that these patients would be covered by Medicare. Therefore, there were 18 289 417 Medicaid-covered and uninsured patient discharges in the sample (10 855 111 [59.4%] in Medicaid expansion states; 7 434 306 [40.6%] in nonexpansion states). Baseline demographic characteristics of study participants are shown in Table 1. The mean (SD) age in expansion states and nonexpansion states was 43.6 (11.7) years and 43.1 (11.6) years, respectively. Overall, in expansion states there were 6 336 747 discharges (58.4%) among female patients and 4 779 860 (44.0%) among White patients. In nonexpansion states, there were 4 440 908 discharges (59.7%) among female patients and 3 332 668 (44.8%) among White patients. At baseline, among discharges for patients aged 26 to 64 years in the expansion states, the mean (SD) proportion of uninsured or Medicaid-insured discharges at a SNH was higher among Black patients (38.6% [12.1]) and Hispanic patients (39.2% [10.5]) relative to White patients (22.6% [6.7]), and in zip codes with higher poverty levels (Table 2).

**Table 1.** Baseline Demographic Characteristics of Study Participants^a^

Characteristic	Patients, No. (%)					
	Medicaid expansion states^b^			Medicaid nonexpansion states^c^		
	All ages	Age 26-64 y	Age ≥65 y	All ages	Age 26-64 y	Age ≥65 y
States, No.	11	11	11	6	6	6
Discharges, 2012-2017, No.	35 816 145	10 855 111	24 961 034	24 816 608	7 434 306	17 382 302
Age, mean (SD), y	67.5 (18.4)	43.6 (11.7)	77.8 (8.5)	67.1 (18.2)	43.1 (11.6)	77.2 (8.3)
Female patients	20 154 499 (56.3)	6 336 747 (58.4)	13 817 752 (55.4)	14 016 152 (56.5)	4 440 908 (59.7)	9 575 302 (55.1)
Male patients	15 661 646 (43.7)	4 518 364 (41.6)	11 143 282 (44.6)	10 800 456 (43.5%)	2 993 398 (40.3)	7 807 058 (44.9)
Race/ethnicity						
White, non-Hispanic	23 479 472 (65.5)	4 779 860 (44.0)	18 699 612 (74.9)	15 955 076 (64.5)	3 332 668 (44.8)	12 662 408 (72.8)
Black, non-Hispanic	4 261 400 (11.9)	2 117 549 (19.5)	2 143 851 (8.6)	4 202 546 (16.9)	2 033 652 (27.4)	2 168 894 (12.5)
Hispanic	5 043 311 (14.1)	2 796 244 (25.8)	2 247 067 (9.0)	3 599 465 (14.5)	1 668 906 (22.4)	1 930 559 (11.1)
Other, including Asian	3 031 962 (8.5)	1 161 458 (10.7)	1 870 504 (7.5)	1 019 521 (4.1)	399 080 (5.4)	620 441 (3.6)
Payer						
Medicaid	9 866 291 (85.5)	9 312 376 (85.8)	Medicare coverage presumed	4 523 951 (58.6)	4 363 442 (58.7)	Medicare coverage presumed
Uninsured	1 674 676 (14.5)	1 542 735 (14.2)		3 197 888 (41.4)	3 071 864 (41.3)	

^a^ The study cohort included hospitalizations for those Medicaid-covered and uninsured individuals aged 26 to 64 years, and all hospitalizations for those aged 65 years and older. Patients 65 years and older were presumed to have Medicare coverage.

^b^ Medicaid expansion states were Arkansas, Arizona, California, Colorado, Iowa, Illinois, Kentucky, New Jersey, New York, Oregon, and Pennsylvania.

^c^ Medicaid nonexpansion states were Florida, Georgia, North Carolina, Texas, Virginia, and Wisconsin.

**Table 2.** Percentage Point Change in Safety-Net Hospitalizations Associated With Medicaid Expansion^a^

Hospitalizations by population group	Percentage of hospitalizations at a safety-net hospital in the baseline year, mean (SD)				Percentage point change associated with Medicaid expansion by year relative to base year (95% CI)^b^				
	Expansion states		Nonexpansion states		1 y before base year	Year 1 after expansion	Year 2 after expansion	Year 3 after expansion	Year 4 after expansion
	Age 26-64 y	Age ≥65 y	Age 26-64 y	Age ≥65					
All	30.3 (7.4)	11.0 (1.7)	29.3 (5.6)	12.2 (2.0)	1.70 (−1.30 to 4.70)	−0.92 (−1.91 to 0.06)	−0.54 (−3.42 to 2.33)	0.27 (−3.5 to 4.03)	0.74 (−2.85 to 4.33)
Race/ethnicity									
White, non-Hispanic	22.6 (6.7)	8.3 (2.5)	22.2 (6.1)	9.3 (3.3)	0.20 (−1.39 to 1.78)	−0.29 (−2.31 to 1.73)	−0.05 (−2.94 to 2.84)	0.22 (−2.47 to 2.90)	1.34 (−3.09 to 5.78)
Black, non-Hispanic	38.6 (12.1)	23.2 (11.4)	38.4 (10.9)	23.6 (12.0)	0.94 (−3.50 to 5.37)	−0.91 (−2.70 to 0.88)	−0.83 (−5.56 to 3.90)	1.09 (−4.71 to 6.90)	3.21 (−4.10 to 10.51)
Hispanic	39.2 (10.5)	23.2 (8.8)	29.3 (14.6)	14.6 (8.7)	1.80 (−2.20 to 5.80)	−0.76 (−2.67 to 1.15)	−1.24 (−2.93 to 0.45)	−0.48 (−3.57 to 2.60)	1.07 (−2.29 to 4.43)
Poverty, zip code median									
≤5%	18.7 (5.9)	5.2 (2.1)	18.1 (5.9)	6.4 (2.7)	−0.59 (−3.02 to 1.84)	−0.99 (−2.88 to 0.90)	−0.19 (−2.74 to 2.36)	−0.21 (−2.10 to 1.68)	3.09 (−1.05 to 7.23)
>5%-10%	21.4 (6.1)	7.0 (2.8)	20.1 (7.2)	7.8 (3.6)	0.73 (−2.29 to 3.75)	−0.88 (−2.34 to 0.59)	−1.08 (−2.94 to 0.78)	−0.08 (−2.75 to 2.59)	2.75 (−1.87 to 7.37)
>10%-20%	29.3 (10.2)	12.8 (5.5)	27.8 (5.0)	12.6 (3.1)	0.96 (−1.86 to 3.77)	−1.47 (−3.01 to 0.07)	−0.69 (−2.96 to 1.59)	0.02 (−3.27 to 3.31)	0.58 (−2.53 to 3.68)
>20%- 30%	34.8 (11.9)	17.7 (7.8)	36.9 (11.0)	20.0 (8.0)	2.71 (−1.61 to 7.02)	−0.65 (−1.66 to 0.35)	−0.35 (−3.01 to 2.30)	0.86 (−2.80 to 4.53)	1.68 (−2.45 to 5.80)
>30%	47.2 (16.3)	29.4 (13.6)	46.3 (13.8)	26.3 (7.9)	1.07 (−0.99 to 3.12)	1.02 (−1.75 to 3.79)	0.91 (−3.91 to 5.72)	0.88 (−4.21 to 5.97)	2.17 (−4.47 to 8.81)

^a^ Safety-net hospitalizations in this table refer to the hospitalizations covered by Medicaid or without insurance. The states included in the study are 11 expansion states (Arkansas, Arizona, California, Colorado, Iowa, Illinois, Kentucky, New Jersey, New York, Oregon, and Pennsylvania) and 6 nonexpansion states (Florida, Georgia, North Carolina, Texas, Virginia, and Wisconsin). In Pennsylvania, expansion occurred on January 1, 2015, and in the remaining 10 states on January 1, 2014. We defined baseline year as 2014 for Pennsylvania and 2013 for all other states.

^b^ Relative year was defined as the number of years before and after expansion using the base year as the reference. For example, in states other than Pennsylvania, 2012 was defined as 1 year before the base year and 2014 as 1 year after the base year. In Pennsylvania, 2013 were defined as 1 year before base year and 2015 as 1 year after base year. A 3-way difference-in-differences model with an event study specification with state-level fixed effects was estimated. The estimates of the change associated with Medicaid expansion are coefficients of the interaction between indicators of expansion state, age group, and relative year. Confidence intervals are based on clustering at the state level. Each row represents the estimates from a separate regression. For each regression, the analytic data consisted of observations formed by aggregation of all hospitalizations for the specific population group by state, year-quarter, and the 2 age groups (ie, 26-64 years and ≥65 years). The outcome measure was the proportion of hospitalizations to a safety-net hospital. The average proportion (%) for each population group in the baseline year are reported separately for the expansion and nonexpansion states.

The observed trend in the percentage of uninsured or Medicaid-insured hospitalizations at an SNH showed no systematic changes in the expansion and nonexpansion states. We found no significant change in uninsured or Medicaid-insured hospitalizations at an SNH associated with Medicaid expansion. Likewise, we found no significant change among all subgroups by race/ethnicity and zip code–level poverty (Table 2).

## Discussion

In this study, hospital utilization patterns suggested that there was socioeconomic, racial, and ethnic segregation in US SNHs. Counter to our hypothesis, increased insurance coverage in ACA Medicaid expansion states did not lead to changes in the hospitals where patients with lower socioeconomic status received care and did not decrease racial and ethnic segregation. It is possible that patients are satisfied with the care they receive and benefit from services that may be unavailable in other settings, such as assistance with insurance, interpretation, and case management. However, it is also possible that the persistence of structural racism and residential segregation prevents patients from transferring care to non-SNHs. A limitation of this study is that unmeasured state policy changes may explain our findings. Extending health insurance coverage alone appears insufficient to reduce hospital segregation by race/ethnicity or socioeconomic status. Future research should identify factors that underlie the use of SNHs among patients with low income.

## Notes

Supplement. **eMethods.** Supplementary Methods **eFigure.** Study States by Expansion Status
