# Supplementary material for: Changes in Hospitalizations at US Safety-Net Hospitals Following Medicaid Expansion
Source: JAMA Netw Open. 2021 Jun 30;4(6):e2114343. doi: 10.1001/jamanetworkopen.2021.14343 (PMC8246310; doi:10.1001/jamanetworkopen.2021.14343)
Supplement: Supplement. — eMethods. Supplementary Methods eFigure. Study States by Expansion Status [file jamanetwopen-e2114343-s001.pdf]

Supplemental Online Content

Lasser KE, Liu Z, Lin MY, Paasche-Orlow MK, Hanchate A. Changes in hospitalizations at US safety-net hospitals following Medicaid expansion. *JAMA Netw Open.* 2021;4(6):e2114343. doi:10.1001/jamanetworkopen.2021.14343

eMethods. Supplementary Methods

eFigure. Study States by Expansion Status

This supplemental material has been provided by the authors to give readers additional information about their work.

© 2021 Lasser KE et al. *JAMA Network Open.*

eMethods. Supplementary Methods

Informed Consent of Study Participants
Institutional Review Boards at Wake Forest and Boston University Schools of Medicine granted an exemption of informed consent.

Data Sources
We obtained comprehensive state inpatient discharge records from 1/1/2012 to 12/31/2017 from eleven states that expanded Medicaid on or prior to 1/1/2015 (Arkansas, Arizona, California, Colorado, Iowa, Illinois, Kentucky, New Jersey, New York, Oregon and Pennsylvania) and six states that did not expand Medicaid (Florida, Georgia, North Carolina, Texas, Virginia and Wisconsin; see **eFigure**). For some states, we used individual state inpatient discharge data from the Agency of Healthcare Research & Quality. For other states, we applied directly to the individual state agencies (e.g. the California Office of Statewide Health Planning and Development, the Illinois Department of Public Health, the Pennsylvania Health Care Cost Containment Council, the Texas Department of State Health Services and the Virginia Health Information) to obtain the data.[7,8] We selected states based on population size, geographic location, completeness of race/ethnicity data (see eTable 1) and data availability. These states accounted for 62.9% of the national population in 2012. The state discharge data contain all individual hospitalization discharge records for all payers from all short-term acute care hospitals with the exception of federally-owned hospitals. We merged these records with zip code-level census population data from the 2010 Decennial Census and poverty rate data (2008-2012) from the American Community Surveys.[9,10]

eTable 1. Share of the study states in the national census population aged 18-64 by race/ethnicity, 2012[a]

State[b]	All[c]	White, non-Hispanic[c]	Black, non-Hispanic[c]	Hispanic[c]
Study States (N=17)				
AZ	2.01%	1.15%	0.09%	0.59%
AR	0.91%	0.67%	0.14%	0.06%
CA	12.43%	5.03%	0.79%	4.55%
CO	1.70%	1.21%	0.07%	0.33%
FL	5.97%	3.29%	0.98%	1.46%
GA	3.17%	1.75%	0.99%	0.27%
IL	4.09%	2.60%	0.59%	0.64%
IA	0.95%	0.84%	0.03%	0.05%
KY	1.38%	1.19%	0.12%	0.04%
NJ	2.83%	1.61%	0.38%	0.54%
NY	6.38%	3.61%	0.96%	1.19%
NC	3.09%	2.01%	0.68%	0.25%
OR	1.26%	0.99%	0.03%	0.14%
PA	4.02%	3.17%	0.45%	0.25%
**State[b]**	**All[c]**	**White, non-Hispanic[c]**	**Black, non-Hispanic[c]**	**Hispanic[c]**
TX	8.19%	3.73%	1.01%	2.99%
VA	2.67%	1.71%	0.52%	0.23%
WI	1.80%	1.51%	0.12%	0.10%
All study states (N=17)	62.87%	36.09%	7.95%	13.68%
Non-Study States (N=33)				
AL	1.51%	1.00%	0.41%	0.06%

AK	0.25%	0.17%	0.01%	0.01%
CT	1.15%	0.80%	0.12%	0.16%
DE	0.29%	0.19%	0.06%	0.02%
DC	0.23%	0.09%	0.10%	0.02%
HI	0.56%	0.16%	0.01%	0.06%
ID	0.48%	0.41%	0.00%	0.05%
IN	2.04%	1.67%	0.19%	0.12%
KS	0.90%	0.70%	0.06%	0.09%
LA	1.45%	0.88%	0.46%	0.07%
ME	0.42%	0.40%	0.01%	0.01%
MD	1.92%	1.03%	0.58%	0.17%
MA	2.17%	1.64%	0.15%	0.22%
MI	3.12%	2.40%	0.45%	0.13%
MN	1.70%	1.42%	0.10%	0.08%
MS	0.92%	0.53%	0.35%	0.03%
MO	1.89%	1.52%	0.23%	0.07%
MT	0.32%	0.28%	0.00%	0.01%
NE	0.57%	0.47%	0.03%	0.05%
NV	0.89%	0.48%	0.08%	0.23%
NH	0.43%	0.39%	0.01%	0.01%
NM	0.65%	0.26%	0.01%	0.30%
ND	0.22%	0.20%	0.00%	0.01%
OH	3.62%	2.94%	0.46%	0.11%
OK	1.22%	0.85%	0.10%	0.11%
RI	0.34%	0.26%	0.02%	0.04%
**State[b]**	**All[c]**	**White, non-Hispanic[c]**	**Black, non-Hispanic[c]**	**Hispanic[c]**
SC	1.48%	0.95%	0.42%	0.08%
SD	0.26%	0.22%	0.00%	0.01%
TN	2.03%	1.53%	0.35%	0.09%
UT	0.86%	0.69%	0.01%	0.11%
VT	0.20%	0.19%	0.00%	0.00%
WA	2.27%	1.64%	0.10%	0.24%
WV	0.58%	0.54%	0.02%	0.01%
WY	0.18%	0.16%	0.00%	0.02%
All non-study states (N=33 & DC)	37.13%	27.04%	4.90%	2.81%
All states (N=50 & DC)	100.00%	63.13%	12.86%	16.49%
Count of population	201,136,985	126,975,739	25,861,075	33,163,609

[a] U.S. Census Bureau (2020). State Population Totals and Components of Change: 2010-2019. Washington, DC, U.S. Census Bureau.[10]
[b] The shaded states are the non-expansion states in this study.
[c] Each cell represents the share (%) of the cohort out of the national count of population aged 18-64 in 2012 (201,136,985). Therefore, the overall share of the Hispanic population in the study states is obtained by the ratio between the cell figure for All study states (13.68%) and for All states (16.49%), which is 83.0%.

Reporting Race/ethnicity

© 2021 Lasser KE et al. *JAMA Network Open.*

We analyzed race data in order to capture unmeasured social factors (e.g. structural racism, racial discrimination)[11] and to identify disparities in utilization of safety-net hospitals. While race is not routinely self-identified in our data, race and ethnicity are separately identified in virtually all the states, the preferred approach.[12] We used the combined race/ethnicity indicator developed by AHRQ.[13] Across all 17 states, the percent of observations having missing data on race/ethnicity 2012-2016 ranged from 0 to 5.8% with a median of 1.7%.

Definition of Safety-net Hospitals

As in a prior study,[14] we defined safety-hospitals based on the percentage of hospitalizations of Medicaid patients and patients whose insurance was "self-pay," "indigent," and "hospital responsibility." We calculated the percentage of hospitalizations in each hospital that fell into these groups in 2012, defining safety-net hospitals as hospitals in the highest quartile. We weighted hospitals according to size.

© 2021 Lasser KE et al. *JAMA Network Open.*

eFigure 1. Study states by expansion status[a]

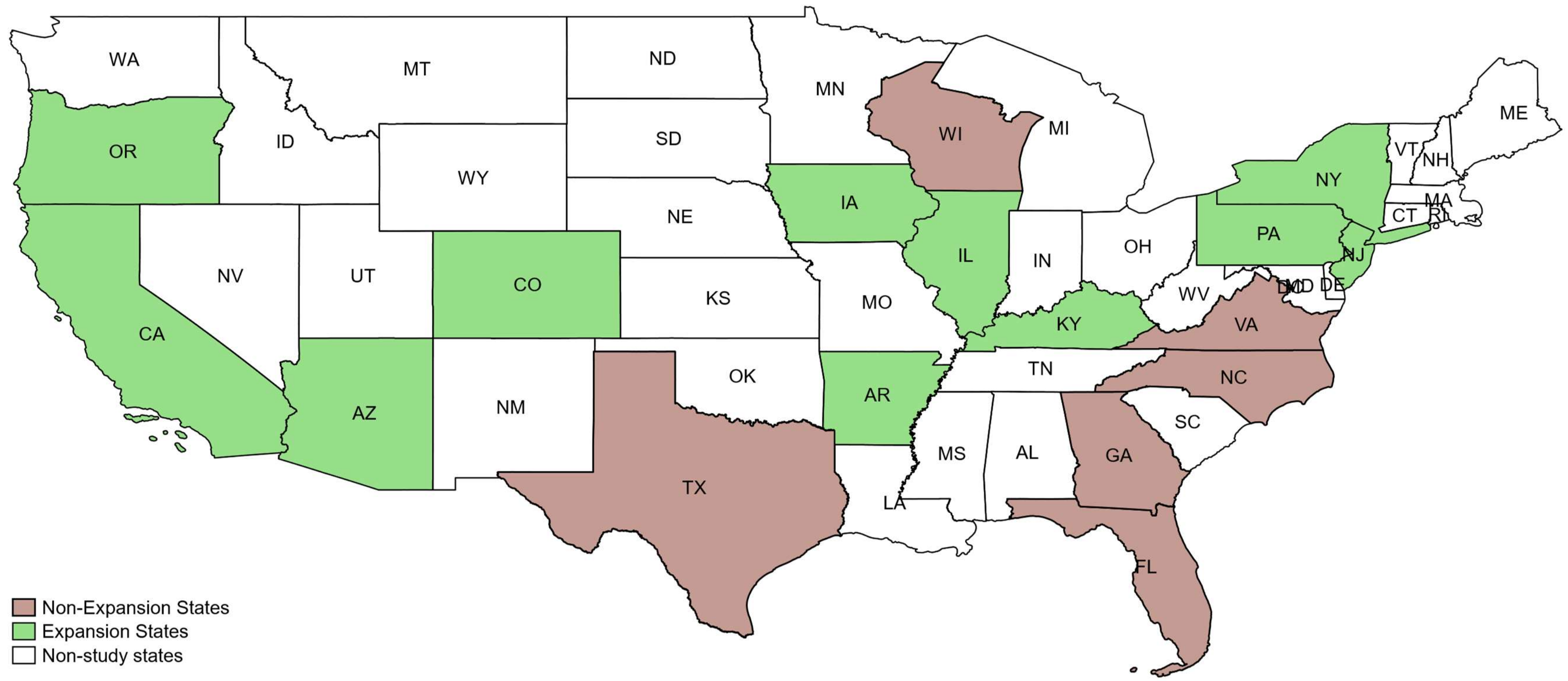

[a]Medicaid expansion status was as of 9/30/2015. Of the 11 expansion states, 10 expanded on 1/1/2014 and Pennsylvania expanded on 1/1/2015.

© 2021 Lasser KE et al. *JAMA Network Open.*
